# Confounders of mortality and hospitalization rate calculations for profit and nonprofit dialysis facilities: analytic augmentation

**DOI:** 10.1186/1471-2369-15-121

**Published:** 2014-07-21

**Authors:** Steven M Brunelli, Steven Wilson, Mahesh Krishnan, Allen R Nissenson

**Affiliations:** 1 DaVita Clinical Research, 825 South 8th Street, Suite 300, Minneapolis, Minnesota 55404, USA; 2 DaVita Healthcare Partners Inc, Denver, Colorado, USA; 3 David Geffen School of Medicine at University of California Los Angeles, Los Angeles, California, USA

## Abstract

**Background:**

Patient outcomes have been compared on the basis of the profit status of the dialysis provider (for-profit [FP] and not-for-profit [NFP]). In its annual report, United States Renal Data System (USRDS) provides dialysis provider level death and hospitalization rates adjusted by age, race, sex, and dialysis vintage; however, recent analyses have suggested that other variables impact these outcomes. Our current analysis of hospitalization and mortality rates of hemodialysis patients included adjustments for those used by the USRDS plus other potential confounders: facility geography (end-stage renal disease network), length of facility ownership, vascular access at first dialysis session, and pre-dialysis nephrology care.

**Methods:**

We performed a provider level, retrospective analysis of 2010 hospitalization and mortality rates among US hemodialysis patients exclusively using USRDS sources. Crude and adjusted incidence rate ratios (IRRs) were calculated using the 4 standard USRDS patient factors plus the 4 potential confounders noted above.

**Results:**

The analysis included 366,011 and 34,029 patients treated at FP and NFP facilities, respectively. There were statistical differences between the cohorts in geography, facility length of ownership, vascular access, and pre-dialysis nephrology care (p < 0.001), as well as age (p < 0.01), race (p < 0.001), and vintage (p < 0.001), but not sex (p = 0.12). When using standard USRDS adjustments, hospitalization and mortality rates for FP and NFP facilities were most disparate, favoring the NFP facilities. Rates were most similar between providers when adjustments were made for each of the 8 factors. With the FP IRR as the referent (1.0), the hospitalization IRR for NFP facilities was 1.00 (95% confidence interval [CI] 0.97-1.02; p = 0.69), while the NFP mortality IRR was 1.01 (95% CI 0.97-1.05; p = 0.64).

**Conclusions:**

These data suggest there is no difference in mortality and hospitalization rates between FP and NFP dialysis clinics when appropriate statistical adjustments are made.

## Background

Since the founding of National Medical Corporation, the first for-profit dialysis chain, in the early 1970’s there has been scrutiny of the level of care provided by commercial dialysis companies. In fact, United States Renal Data System (USRDS) analytic files have a flag identifying a provider as either not-for-profit (NFP) or for-profit (FP) in order to facilitate comparisons. Over the years, a number of comparative analyses of FP versus NFP providers have been published [1-5]. Each year, the USRDS Annual Data Report includes a dialysis provider-level comparison of mortality and hospitalization rates between FP and NFP facilities that is adjusted for age, race, sex, and dialysis vintage [6]. More recently, it has been observed that patients dialyzing with FP and NFP facilities differ on the basis of other key patient and non-patient factors—such as pre-dialysis nephrology care, length of ownership for dialysis clinics, geography or location of dialysis clinics, and vascular access at dialysis initiation—that themselves are important determinants of patient outcomes [1,7-14]. (Mahesh Krishnan, T Christopher Bond, Steven Brunelli, Allen Nissenson. Impact of Potential Confounders on Comparisons Between United Stated For-Profit and Nonprofit Dialysis Providers, Nephrol Dial Transplant. (2013) 28 (suppl 1): i258-i270.) Because NFP facilities tend to have more favorable distributions of these factors, it is possible that prior published comparisons of outcomes between FP and NFP facilities have biases that affect the results. With the advent of sophisticated analytical techniques, more recent provider-level analyses have attempted to account for confounding derived from differences that exist between dialysis provided at FP versus NFP facilities [15].

The purpose of our current investigation was to characterize case mix differences between FP and NFP facilities and to estimate the associations of profit status with mortality and hospitalization rates adjusted for traditional and non-traditional confounders so as to provide less biased comparisons. In order to minimize ascertainment bias, all study data were taken from USRDS Standard Analytical Files.

## Methods

We performed a provider level, retrospective, observational analysis of hospitalization and mortality rates among patients receiving hemodialysis at US dialysis facilities during 2010. Study data were taken from the 2010 USRDS Standard Analytical Files, which are available for analysis by request with associated data access fees. In USRDS data, hospitalizations are identified from Medicare Part A claims; therefore study inclusion was limited to patients with Medicare Part A primary insurance. The primary exposure of interest was dialysis facility ownership status of each Medicare beneficiary’s treating dialysis organization (ie, FP versus NFP). We calculated the study outcomes according to FP and NFP center status as rates expressed in hospitalizations per patient-year at risk or deaths per 100 patient-years at-risk. Patients were considered at-risk from 1 January 2010, or from the date of ESRD enrollment during calendar year 2010, until death or censoring for discontinuation of dialysis or end of study period (31 December 2010). Patient hospitalizations were drawn from the USRDS Institutional Claims Files over the same period. Covariate data were likewise derived from USRDS Standard Analytical Files. Confounders considered were those variables commonly used to adjust FP versus NFP comparisons (age, sex, race, and dialysis vintage), as well as the ESRD network (as a measure of geography), vascular access modality at first dialysis treatment (central venous catheter [CVC], arteriovenous fistula [AVF], arteriovenous graft [AVG]); and pre-dialysis nephrology care (none; < 6 months, 6–12 months, > 12 months). Facility length of ownership was another potential confounder considered for analysis and was identified by examining each facility’s ascribed organization over historically successive USRDS Facility Reports (2007–2010).

The retrospective nature of this analysis made it exempt from Institutional Review Board or Ethics Committee approval by the DaVita clinical operations team.

Dialysis chains were described according to characteristics of constituent patients at baseline (1 January 2010 or date of ESRD enrollment). Continuous patient variables are presented as means with standard deviations, and compared using t-tests. Categorical variables were described as frequencies and proportions and compared using chi-square tests.

Crude incidence rates were calculated as the number of events divided by the cumulative at-risk time. Modeled rates and 95% confidence intervals (CIs) were based on a negative binomial distribution due to observed over-dispersion of Poisson models. Unadjusted incidence rate ratios (IRRs) were estimated using exposure-only negative binomial models. Adjusted IRRs were estimated by sequential addition of covariate terms for baseline patient characteristics. Because first dialysis access type and the flag indicating presence or absence of pre-dialysis nephrology care were missing in some instances, analyses adjusted for these factors were limited to patients with available data for the corresponding variable. For each outcome, 5 unique regression models were fit. In the first model, IRRs were adjusted as per the USRDS analyses accounting for age, sex, race, and dialysis vintage (Model 1, the USRDS standard model). For the second model (Model 2), adjustments were made according to Model 1 plus, geographic variations (ESRD network) and facility length of ownership. In Model 3, the factors adjusted in Model 2 were considered in addition to vascular access at first dialysis. Model 4 included factors adjusted for in Model 2 plus pre-dialysis nephrology care. Model 5 adjusted IRR calculations for all 8 of the factors identified, including those for the USRDS standard model (Model 1: age, sex, race, and dialysis vintage) plus ESRDS network, length of facility ownership, vascular access at first dialysis session, and pre-dialysis nephrology care.

In sensitivity analyses, missing data for pre-dialysis nephrology care and vascular access type at first dialysis were multiply imputed. Briefly, a joint multinomial model was used to impute pre-dialysis nephrology care (none; < 6 months, 6–12 months, > 12 months) and first dialysis vascular access (AVF, AVG, CVC, other). Pre-dialysis nephrology care and vascular access type were mutually referential (ie, each predicted the other), and also predicted on the basis of all covariates from Model 5, follow-up time (log transformed), and outcome indicators (using the ICE procedure in Stata 10.0 MP). Five bootstrap replicates were created. Outcome models were fit using the MIM macro in Stata, which accounts for multiplicity of observations across imputation set.

## Results

Dialysis provider level demographic information for the patient populations studied is provided in Table 1. The mean age of patients treated at FP (N = 366,011) and NFP (N = 34,029) dialysis facilities was 62.6 and 62.3 years, respectively (p < 0.01). Mean dialysis vintages were 2.6 and 2.7 years, respectively (p < 0.001). Patients treated at FP facilities were more likely to be white or black, and less likely to be Asian, Native American, or Pacific Islander (p < 0.001). There was no significant gender imbalance between FP and NFP facilities (p = 0.12).

**Table 1:** Comparison of baseline demographic information between patients dialyzing at for-profit versus not-for-profit facilities

	**For-Profit**		**Not-For-Profit**		**P-value**
	**N = 366,011**		**N = 34,029**		
**Continuous variables**	**Mean**	**Standard Deviation**	**Mean**	**Standard Deviation**	
Age (years)	62.6	14.9	62.3	15.2	< 0.01
Vintage (years)	2.6	3.0	2.7	3.2	< 0.001
**Categorical variables**	**N**	**Percent**	**N**	**Percent**	
Male sex	202,771	55.4	19,002	55.8	0.12
Race					< 0.001
White	212,799	58.1	19,389	57.0	
Black	131,554	35.9	11,712	34.4	
Asian	16,120	4.4	1,889	5.6	
Native American/Pacific Islander	4,390	1.2	943	2.8	
Other	1,055	0.3	91	0.3	
Unknown	93	0.03	5	0.01	

Additional patient and dialysis center variables are presented in Table 2. Length of ownership was significantly longer for NFP versus FP facilities. In the FP population (N = 344,555), 94.1% of centers had an ownership of ≥ 4 years compared to the 94.7% in the NFP population NFP (N = 32,224; p < 0.001). Patients treated at NFP dialysis facilities more frequently received their first dialysis treatment with an AVF (19.7%) compared to those patients in the FP population (17.0%; p < 0.001), while individuals treated at FP dialysis facilities were more frequently first dialyzed with a CVC in place (78.0%) than NFP (75.6%; p < 0.001). Length of pre-dialysis nephrology care received by patients also differed significantly between study populations (p < 0.001). In NFP dialysis facilities, 37.2% of patients received > 12 months of pre-dialysis care compared to 30.4% of patients who dialyzed at FP centers. Patients receiving dialysis at FP facilities went without any pre-dialysis nephrology care more frequently than patients who dialyzed at NFP facilities (30.3% versus 25.2%, respectively). Table 2 also provides the number of facilities operating during the year 2010 in each of the 18 USRDS ESRD networks. Large disparities between the study populations related to geographical distribution are demonstrated, particularly in networks 10 (Illinois), 14 (Texas), 16 (Northwest region), and 18 (central and southern California), where the proportion of clinics were heavily skewed towards either FP or NFP status.

**Table 2:** Comparison of additional potential confounding factors between for-profit and not-for-profit facilities

	**For-Profit N = 366,011**		**Not-For-Profit N = 34,029**		**P-value**
	**N**	**Percent**	**N**	**Percent**	
**Facility length of ownership**					< 0.001
2 years	9,106	2.5	888	2.6	
3 years	12,350	3.4	917	2.7	
≥ 4 years	344,555	94.1	32,224	94.7	
**Vascular access at first treatment**					< 0.001
Catheter	208,016	78.0	18,182	75.6	
Fistula	45,243	17.0	4,731	19.7	
Graft	10,999	4.1	950	4.0	
Other	2,605	1.0	174	0.7	
**Length of predialysis care**					< 0.001
> 12 months	76,905	30.4	8,915	37.2	
6-12 months	62,785	24.8	5,868	24.4	
< 6 months	36,510	14.4	3,165	13.2	
None	76,662	30.3	6,050	25.2	
**ESRD network region**					< 0.001
1	11,036	3.0	629	1.9	
2	15,084	4.1	3,339	9.8	
3	8,373	2.3	662	2.0	
4	16,002	4.4	1,565	4.6	
5	21,864	6.0	791	2.3	
6	37,720	10.3	4,804	14.1	
7	26,229	7.2	1,110	3.3	
8	22,549	6.2	3,220	9.5	
9	28,838	7.9	2,259	6.6	
10	16,832	4.6	76	0.2	
11	18,171	5.0	3,223	9.5	
12	12,401	3.4	2,290	6.7	
13	17,128	4.7	614	1.8	
14	39,625	10.8	284	0.8	
15	17,924	4.9	2,201	6.5	
16	8,122	2.2	2,963	8.7	
17	16,108	4.4	3,330	9.8	
18	32,005	8.7	669	2.0	

**Figure 1:**
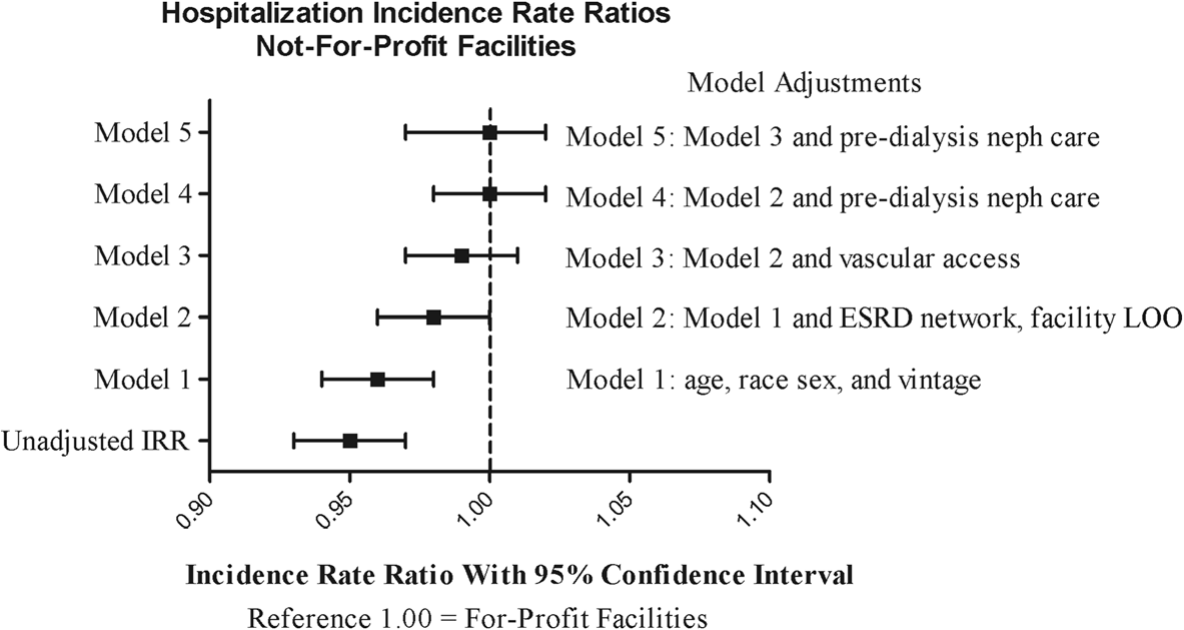
**Hospitalization incidence rate ratios at not-for-profit versus for-profit facilities.** Abbreviations: ESRD, end-stage renal disease; LOO, length of ownership.

Provided in Table 3 are the hospitalization rates for the FP and NFP populations. The crude unadjusted rate for hospitalization was greater in the FP population (1.66 hospitalizations per patient-year) than the NFP population (1.59 hospitalizations per patient-year), corresponding to a crude IRR for NFP of 0.95 (95% CI, 0.93-0.97) versus FP facilities (IRR 1.0 referent). Estimates were nearly identical when adjusted for the standard array of covariates (Model 1): IRR 0.96 (95% CI, 0.94-0.98). With sequential addition of additional covariates, the protective association of NFP was incrementally attenuated and no longer statistically significant (Table 3, Figure 1). In the fully adjusted model (Model 5), the NFP facility IRR for hospitalization was 1.00 (95% CI, 0.97-1.02; p = 0.69).

**Table 3:** Comparison of hospitalization rates between for-profit and not-for-profit facilities

	**Incidence Rate Ratio**		
	**For-Profit**	**Not-For-Profit**	**P-value**
	**N = 366,011**	**N = 34,029**	
	**PtY = 290,995**	**PtY = 27,297**	
**Hospitalization rates**			
Hospital admissions	482,922	43,362	-
Crude rate per year	1.66	1.59	-
Modeled rate per year (95% CI)^a^	1.83 (1.82-1.84)	1.74 (1.71-1.77)	-
Unadjusted IRR (95% CI)^a^	1 (reference)	0.95 (0.93-0.97)	< 0.001
**Model 1 (standard model)**^**a**^**:** IRR (95% CI) adjusted for age, race, sex, vintage [N = 400,040]	1 (reference)	0.96 (0.94-0.98)	< 0.001
**Model 2**^**a**^**:** IRR (95% CI) standard model + ESRD network, facility LOO [N = 400,040]	1 (reference)	0.98 (0.96-1.00)	0.02
**Model 3**^**a**^**:** IRR (95% CI) standard model + ESRD network, facility LOO, first access [N = 290,900]	1 (reference)	0.99 (0.97-1.01)	0.25
**Model 4**^**a**^**:** IRR (95% CI) standard model + ESRD network, facility LOO, pre-dialysis nephrology care [N = 276,680]	1 (reference)	1.00 (0.98-1.02)	0.89
**Model 5**^**a**^**:** IRR (95% CI) standard model + ESRD network, facility LOO, first access, pre-dialysis nephrology care [N = 252,270]	1 (reference)	1.00 (0.97-1.02)	0.69

Abbreviations: CI, confidence interval; ESRD, end-stage renal disease; IRR, incident rate ratio; LOO, length of ownership; PtY, patient-years.

^a^Negative binomial model.

**Figure 2:**
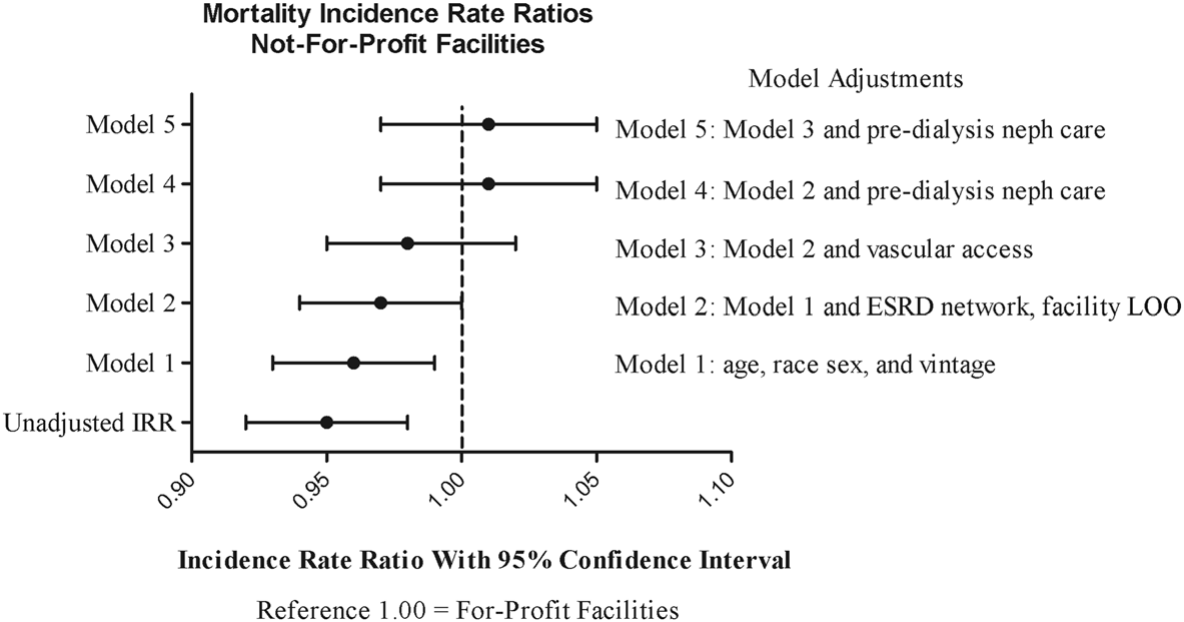
**Mortality incidence rate ratios at not-for-profit versus for-profit facilities.** Abbreviations: ESRD, end-stage renal disease; LOO, length of ownership.

The crude mortality rate in the FP population was 23.1 deaths per 100 patient-years compared to 22.0 deaths per 100 patient-years in the NFP population (Table 4). The unadjusted mortality IRR for the NFP group was found to be 0.95 (95% CI, 0.92-0.98; p = 0.003). Again, estimates were different when adjusted for age, race, sex, and vintage: the NFP IRR was 0.96 (95% CI, 0.93-0.99; p = 0.009), but were incrementally attenuated and lost statistical significance upon additional covariate adjustments (Table 4, Figure 2). In the fully adjusted model (Model 5), the NFP IRR for death was 1.01 (95% CI, 0.97-1.05; p = 0.64).

**Table 4:** Comparison of mortality rates between for-profit and not-for-profit facilities

	**Incidence Rate Ratio**		
	**For-Profit**	**Not-For-Profit**	**P-value**
	**N = 366,011**	**N = 34,029**	
	**PtY = 290,995**	**PtY = 27,297**	
**Mortality rates**			
Deaths	59,611	5,366	-
Crude rate per 100 patient-years	20.5	19.7	-
Modeled rate per 100 patient-years (95% CI)^a^	23.1 (22.7-23.4)	22.0 (21.3-22.7)	-
Unadjusted IRR (95% CI)^a^	1 (reference)	0.95 (0.92-0.98)	0.003
**Model 1 (standard model)**^**a**^**:** IRR (95% CI) adjusted for age, race, sex, vintage [N = 400,040]	1 (reference)	0.96 (0.93-0.99)	0.009
**Model 2**^**a**^**:** IRR (95% CI) standard model + ESRD network, facility LOO [N = 400,040]	1 (reference)	0.97 (0.94-1.00)	0.03
**Model 3**^**a**^**:** IRR (95% CI) standard model + ESRD network, facility LOO, first access [N = 290,900]	1 (reference)	0.98 (0.95-1.02)	0.40
**Model 4**^**a**^**:** IRR (95% CI) standard model + ESRD network, facility LOO, pre-dialysis nephrology care [N = 276,680]	1 (reference)	1.01 (0.97-1.05)	0.52
**Model 5**^**a**^**:** IRR (95% CI) standard model + ESRD network, facility LOO, first access, pre-dialysis nephrology care [N = 252,270]	1 (reference)	1.01 (0.97-1.05)	0.64

Abbreviations: CI, confidence interval; ESRD, end-stage renal disease; IRR, incident rate ratio; LOO, length of ownership; PtY, patient-years.

^a^Negative binomial model.

Sensitivity analyses were conducted in which missing values for pre-dialysis nephrology care and first vascular access type were imputed. In these analyses, there was no statistically significant difference in the risk for either hospitalization or death: fully adjusted IRRs for NFP were 0.98 (95% CI, 0.97-1.00; p = 0.07) and 0.97 (95% CI, 0.94-1.00; p = 0.09), respectively.

## Discussion

In the past, USRDS comparisons between FP and NFP dialysis providers for rates of hospitalization and mortality have been adjusted for age, sex, race, and dialysis vintage [6]. In our current analysis, the magnitude of the differences between the FP and NFP populations for these patient factors were clinically trivial, yet statistically significant: age, 0.3 year; vintage, 0.1 year; sex, 0.4 percentage point; and race, < 2 percentage points for any given category. Here, rate estimates adjusted for these factors (Model 1) are not substantively different from unadjusted estimates. Consistent with recent literature, we have shown more substantive differences in pre-dialysis nephrology care (5–7 percentage points) [7,8], vascular access at first dialysis (2.5 percentage points) [7,16], and geographic region [17,18]. These factors, though not under the control of the dialysis facility, are potent determinants of patient outcomes. Thus, considering these factors together with age, sex, race, and dialysis vintage renders a potential effect of profit status on patient outcomes undetectable. Our results likely explain the lack of association between profit status and patient outcomes identified in the single previous report that used instrumental variables analysis [15]. Instrumental variables analysis adjusts for both measured and unmeasured confounding. The unmeasured confounding in the previous study likely included the specific differences we identified in facility geography, length of facility ownership, vascular access at first dialysis session, and pre-dialysis nephrology care [15].

A dramatic finding of this study is the large disparity between FP and NFP clinic geography within the US, which is known to significantly impact patient mortality [13]. While the variables of vascular access at first dialysis session and pre-dialysis nephrology care were adjusted for in this study, clinic geography is likely to have other important impacts on health care outcomes. For example, there are fewer nephrologists available for consultation in rural areas [19], and early pre-dialysis care with a specialist positively impacts dialysis patient survival [20,21], as well as affording educated decisions for selection of modality and vascular access [22]. Currently unpublished data demonstrate that the large FP US dialysis organizations disproportionately serve rural and high poverty urban areas compared to other providers, [Fadi Almachraki et al., 2014 manuscript in preparation] further confounding comparative analyses between large and small providers and FP and NFP facilities.

Our results beg the question: why are these additional variables confounding analyses of mortality and hospitalization rates between clinic categories? One reason may be referral patterns of health care providers who directly influence where patients receive care. Health care referral patterns are affected by dialysis clinic geography and arbitrary factors such as the availability of private or public transportation to clinics, and hours of clinic operation. Health care provider beliefs, insurance provider policies, and institutional practices are all likely to impact whether patients are referred for pre-dialysis care to nephrologists, who—according to beliefs, policies, and practices-would recommend patients for surgery to receive AVF placement prior to dialysis initiation. Clinic geography, which in our analysis was widely varied across FP and NPF providers, probably affects patient outcomes, as isolated clinics in either urban or rural locations may be the only available dialysis facility in a given region, and patients have no choice of provider to patronize. Moreover, NP and NFP dialysis organizations have fundamentally different risk tolerance to drive growth by either *de novo* clinic development or acquisition of existing dialysis clinics. Potential differences in acceptable economic risk are probably heavily influenced by geographic area, including the density of patients with ESRD requiring dialysis treatment within a given area, and the available facilities within the area. This “growth” variable directly impacts length of clinic ownership, which we have demonstrated as affecting estimates for hospitalization and mortality rates.

In addition to varying economic risk tolerance, there are probably real philosophical differences between FP and NFP dialysis providers; however these differences are not necessarily negative. For instance, there may be a perception that quality concessions can be used to maximize profits. However, in actuality the opposite is true. The Centers for Medicare and Medicaid Services Quality Incentive Program penalizes facilities up to 2% of all Medicare remuneration for failure to meet quality metrics [23], and publicizes financial penalties levied on poor-performing dialysis clinics, creating both direct and indirect incentives for good care. Moreover, considering that facility census is the chief economic driver, any sacrifice in quality that leads to missed treatments (eg, due to hospitalization), death, or transfer of care away from the provider organization are economically counter-productive. Furthermore, profit motives may spur clinical innovations such CathAway [24], Incident Management of Patients, Actions Centered on Treatment (IMPACT) [25], and RightStart [26], which positively affect outcomes for hemodialysis patients. The integration of ESRD patient care with other aspects of healthcare have also been facilitated by the FP LDOs including surgical centers for vascular access and endovascular care (LifeLine, Ultracare), specialty pharmacies for dialysis patients (DaVita Rx [27], Fresenius Rx) and patient-focused programs for diabetes disease management (Village Health StepAhead). Although there are clear financial incentives to such programs, all fundamentally benefit patient care.

While the FP category of dialysis providers in the US is dominated by 2 LDOs, the NFP category is dominated by one LDO, such that its business practices and history alone influence the entire category. A post-hoc analysis (data not shown) demonstrated that this single NFP LDO had superior hospitalization and mortality rates to FP centers, whereas the remainder of NFP centers collectively performed inferiorly. Unknown factors, related or possibly unrelated to the quality of care, may be driving the differences in outcomes between this one NFP LDO and other NFP dialysis chains.

We recognize a limitation of this study is its observational design, which can only measure associations between exposure and the outcomes studied. Although we attempted to adjust analyses for a robust vector of covariates that characterizes salient differences between patient populations served by FP versus NFP dialysis organizations, we acknowledge that there may be residual confounding on the basis of variables not considered. However, it is unlikely that a randomized trial allocating patients to FP versus NFP centers will ever be conducted to definitively assess causal effects.

## Conclusions

It is our belief that these data demonstrate a need for additional adjustments to be made to analytics comparing rates of hospitalization and mortality between dialysis providers or units. Omission of these factors in analytic adjustments can result in conclusions that may not accurately reflect differences in patient outcomes. Since a randomized prospective trial with regards to profit status of dialysis patients is impractical, we believe that the current study, in addition to other emerging literature, demonstrates that no difference exists in outcomes between dialysis providers stratified by profit status. Furthermore we believe that some heterogeneity exists in all provider types such that broad classification of providers by a single profit status flag may also create unintended and misleading results.

## Competing interests

DaVita Clinical Research is a wholly-owned subsidiary of DaVita HealthCare Partners; authors are employees of Davita HealthCare Partners a large dialysis organization. None of the authors are aware of any non-financial competing interests.

## Authors’ contributions

SW and SMB conducted the statistical analysis. SMB, SW, MK, and ARN conceived of the study, participated in its design, and helped to draft the manuscript. All authors read and approved of the final manuscript.

## Authors’ information

SW is a biostatistician holding a PhD with several years experience in observational analyses of the DaVita Clinical Data Warehouse patient database. SMB, MK, and ARN are each medical doctors trained as nephrologists. SMB, Vice President and Medical Director of DaVita Clinical Research, holds an advanced degree in clinical epidemiology, and has many years experience conducting analyses of the DaVita Clinical Data Warehouse. MK is Vice President of Clinical Research and serves the Office of the Chief Medical Officer at DaVita HealthCare Partners. ARN is the Chief Medical Officer of DaVita HealthCare Partners and Professor Emeritus of Medicine at David Geffen School of Medicine at the University of California, Los Angeles.

## Pre-publication history

The pre-publication history for this paper can be accessed here:

http://www.biomedcentral.com/1471-2369/15/121/prepub
